# Surface and Subsurface Analysis of Stainless Steel and Titanium Alloys Exposed to Ultrasonic Pulsating Water Jet

**DOI:** 10.3390/ma14185212

**Published:** 2021-09-10

**Authors:** Jakub Poloprudský, Alice Chlupová, Ivo Šulák, Tomáš Kruml, Sergej Hloch

**Affiliations:** 1 Institute of Physics of Materials, CAS (Czech Academy of Sciences), 602 00 Brno, Czech Republic; poloprudsky@ipm.cz (J.P.); chlupova@ipm.cz (A.C.); sulak@ipm.cz (I.Š.); 2 Central European Institute of Technology, Institute of Physics of Materials, CAS (Czech Academy of Sciences), 602 00 Brno, Czech Republic; kruml@ipm.cz; 3 The Czech Academy of Sciences, Institute of Geonics, Studentska 1768, 708 00 Ostrava-Poruba, Czech Republic

**Keywords:** initial erosion stage, 316L, Ti6Al4V, eroded surface topography, water volume droplets, dislocation structure

## Abstract

This article deals with the effect of periodically acting liquid droplets on the polished surfaces of AISI 316L stainless steel and Ti6Al4V titanium alloy. These materials were exposed to a pulsating water jet produced using an ultrasonic sonotrode with an oscillation frequency of 21 kHz placed in a pressure chamber. The only variable in the experiments was the time for which the materials were exposed to water droplets, i.e., the number of impingements; the other parameters were kept constant. We chose a low number of impingements to study the incubation stages of the deformation caused by the pulsating water jet. The surfaces of the specimens were studied using (1) confocal microscopy for characterizing the surface profile induced by the water jet, (2) scanning electron microscopy for detailed surface observation, and (3) transmission electron microscopy for detecting the changes in the near-surface microstructure. The surface described by the height of the primary profile of the surface increased with the number of impingements, and was substantially more intense in the austenitic steel than in the Ti alloy. Irregular surface depressions, slip lines, and short cracks were observed in the Ti alloy, whereas pronounced straight slip bands formed in the austenitic steel. The dislocation density near the surface was measured quantitatively, reaching high values of the order of 10^14^ m^−2^ in the austenitic steel and even higher values (up to 3 × 10^15^ m^−2^) in the Ti alloy. The origins of the mentioned surface features differed in the two materials: an intense dislocation slip on parallel slip planes for the Ti alloy and mechanical twinning combined with dislocation slip for the austenitic steel.

## 1. Introduction

Water jet technology has been extensively used for cutting, drilling, and cleaning [1]. Water jet peening, a nonthermal method for surface strengthening, is considered a relatively new advance compared with other applications of water jet technology [2,3]. To enhance the advantages of water jets, two approaches are possible. The first is the addition of solid particles, which are erodents, into the water jet [4]; the accelerated abrasives then wear the material [5]. The second method to increase the efficiency of the water jet is the forced decaying of the continuous water jet into discrete clusters to use the hammer effect [6]. By periodically applying impact pressure in the form of a water droplet, which has higher pressure values than stagnation pressure [7], in a continuous stream, it is possible to erode the material at lower operating pressures of 10–100 MPa [8]. The advantages of pulsating water jets (PWJs) can be grouped into three main areas: first, they are more efficient compared with continuous water jets under the same hydraulic conditions [9]; second, the pressure acts upon the impacted area in a periodic manner, which can be used for surface reinforcement, such as the peening method [10,11]; and third, A PWJ acting without added solid (abrasive) particles can be used in the treatment of structural parts for medical applications [12,13,14,15].

The mechanical interruption of the continuous stream can be achieved by rotating disks [16] or the hybrid modification of the water stream by ultrasonically excited PWJs [17]. Jet decaying using rotating discs with perforations on the perimeter with defined dimensions causes artificial decaying of the jet, which results in the formation of a discontinuous current with a low frequency. The problem of individual water droplets is the moment of inertia at its ends where the circumferential speed is perpendicular to the continuous jet. Part of the energy of the jet is absorbed by the surface of the rotating disk. This erosion damage to the mechanical modulator decreases the lifetime of the device (modulator). An effective method to overcome this issue is to add mechanical vibrating droplets (needle) inside a pressure chamber close to the nozzle outlet. A problem with this method is the short life of the needle [8]. A self-resonating nozzle works using a specially made cavity inside the nozzle. The fluctuations in the flow are created inside the cavity by the interaction of stationary waves in the inlet area and the flow reflected on the outlet side. Another investigated and patented method is the ultrasonic pulsating water jet [18,19]. The device is assembled so that the sonotrode oscillates in the acoustic chamber [18] in the axis and supplies additional momentum to the water under a certain pressure. The created pressure fluctuations are amplified by a transmitting cone, at the end of which is a nozzle. As such, it is possible to achieve a wide range of water droplets with different volumes and velocities [20]. When the feedback oscillation and the natural frequency of the nozzle match, the continuous water jet shatters into a PWJ. The method, which was used in the current study, was developed at the Institute of Geonics AS CR, v. v. i. This method consists of a piezoelectric crystal that is connected to an ultrasonic generator and a sonotrode. Piezoelectric crystals using inverse piezoelectric phenomena transform electrical energy into mechanical energy. Due to the dipole moment, the length of the piezoceramic crystal changes. Crystal starts to vibrate based on the electric impulses introduced by the ultrasonic generator. The vibrations of the piezoelectric crystal then oscillate with the sonotrode. The sonotrode is in contact with pressurized (10–100 MPa) water, where the sonotrode creates pressure fluctuations. Once the pressurized water leaves the nozzle, the pressure fluctuations change into velocity fluctuations, which then split the continuous water current into separate clusters of droplets [9]. However, the pulses are not effective immediately after exiting the nozzle [21]. From the beginning, the pulsating water stream appears to be continuous. Effective decaying occurs for a certain standoff distance, which is a function of the frequency, pressure, acoustic chamber length, and nozzle diameter [19]. Four main damage mechanisms, occurring when the droplet hits the solid surface, have been described [22].

- The first is the direct action. This deformation mode describes how the water hammer effect influences the material during the compressible stage. The mode of deformation depends heavily on the response of the type of treated material to the dynamic and transient nature of this loading.
- The second erosion mechanism is called stress wave propagation. The transient pressure in the compressible stage causes stress waves through solid materials. These stress waves then interact with microscopic material features such as precipitates, grain boundaries, and phase boundaries [7].
- The third erosion mechanism is known as lateral outflow jetting. This mechanism is caused by high-velocity lateral jets produced in the flow stage. This stage is dependent on the surface roughness; when the PWJ interacts with surface asperities, it may lead to plastic deformation, the formation of erosion pits, and crack initiation and propagation.
- The fourth erosion mechanism is called hydraulic penetration. This mechanism covers the interaction of a PWJ with erosion pits and cracks. These surface irregularities cause stress concentrations, which can result in accelerated propagation of the cracks, tunneling under the surface of the material, and upheaval of the material overlaying the cracks [23].

Foldyna et al. [9], using experiments of eroding aluminum with varying process parameters (namely standoff distance), proved the existence of three zones of PWJ action with respect to the standoff distance.

- In the first zone, which is closest to the nozzle, velocity fluctuations do not completely shatter the jet into separate discrete clusters; therefore, the jet is acting continually.
- The second zone is defined by the highest erosion rate, where the jet is striking the surface with well-developed pulses.
- The third zone is specified by the break-up of discrete clusters into small droplets due to aerodynamic drag.

Nag et al. [20] defined two limits for standoff distance based on effective erosion for various process parameters, and especially the length of the acoustic chamber. Hloch et al. [24] more precisely divided pulsating water jets into five distinct areas based on the standoff distance with respect to the jet morphology and eroded depth: incubation, acceleration, culmination, depletion, and termination. In the incubation stage, the PWJ acts similarly to a continuous jet. The length of the incubation stage (distance) depends on supply pressure. The effective distance at which the water droplets capable of generating the impact pressure are formed depends on the hydraulic parameters. In the incubation regime, stagnation pressure prevails over water hammer pressure. In the acceleration regime, velocity fluctuations cause separation of the water clusters; therefore, impact pressure prevails over stagnation pressure. The culmination regime is defined by the formation of discrete water clusters, leading to a repetitive water hammer effect on the impacted surface. The depletion stage shows signs of water wave deconcentration due to aerodynamic drag, which decreases PWJ impact pressure. In the termination stage, i.e., the final stage, monodisperse droplets with lesser mass than the clusters in the acceleration and depletion stage interact with the surface. A further study proved the erosion shift of the grooves in terms of the pressure variability and distribution density of water clusters [8].

Foldyna et al. [25] focused on the effect of increasing the number of impingements on the surface of stainless steel treated with a PWJ. The number of impingements ranged from 2125 to 106,250. Based on the surface roughness, the effects of increasing the number of impacts were separated into three stages: the initial stage marked by the plastic deformation of the impacted material, the second stage characterized by the creation of erosion pits and their merging into a single erosion crater, and the third stage defined by the increasing depth of a single crater. The increase in roughness in the second stage is greater than that in the third stage. A similar effect of increasing the number of impingements was observed as early as the 1970s by Hancox et al. [26] and Thomas et al. [27], who used the wheel and jet apparatus and the number of impingements correlated with the number of wheel turns. Thomas et al. [27] described the stages of dependency of cumulative erosion on the number of impacts on austenitic steel. The first stage was defined by a reduction in the reflectivity of the surface caused by the creation of shallow depressions of the size of 1–10 µm. Further increases in the number of impacts led to an increase in the size and depth of depressions and tilting of the grain boundaries. The next stage was defined by the start of material breakage and coalescence of the small depressions into a groove. The last stage was recognized by the deepening of the groove. These stages can be compared to the stages observed by Foldyna [25].

A PWJ was used as a surface strengthening process in several experiments described in Srivastava et al. [15,28] Lehocka et al. [16], and Srivastava et al. [17]. The process causes local plastic deformation due to the interaction of the waterfront of the water clusters with the material. The deformation induces compressive residual stresses in the surface and subsurface layers. For cyclically loaded structural parts, this layer is beneficial as it delays the initiation of the fatigue cracks and can thus increase the fatigue life in high-cycle areas [15]. In PWJs, the influence of the repeated water hammer effect (i.e., a high number of cycles creating residual stresses) on the damage evolution in the material is strong, which is why one of the goals of this study was to document the evolution of the dislocation structure under the impacted area.

Most researchers studying PWJ technology have focused on the parameters of the process from the technological point of view. From a materials point of view, the changes in the dislocation structure of the surface layer of the treated material are deeply connected to the material’s parameters such as material crystallographic lattice and stacking fault energy. These changes then affect the surface relief of the treated material. We focused on the observation and comparison of the incubation stage of a PWJ applied to Ti6Al4V titanium alloy, which is widely used in medical applications, and to AISI 316L, as a typical austenitic stainless steel. The controlled parameter of the experiment was the number of impingements striking the surface of the materials. We aimed to understand the effect of the number of impingements of water clusters on the evolution of the surface relief, the related dislocation structure created inside the surface and subsurface layers of the sample, and the plastic deformation of the surface. This knowledge could be applied to more accurately tune PWJ parameters (the water cluster distribution for surface strengthening). The main experimental techniques used in our study for this purpose were scanning electron microscopy (SEM), transmission electron microscopy (TEM), and confocal microscopy.

## 2. Experimental Materials and Methods

The experiment consisted of the following steps: material surface polishing, exposure of the surfaces to the periodically acting water droplets under variable time, and wear damage analysis.

### Materials

**Figure 1:**
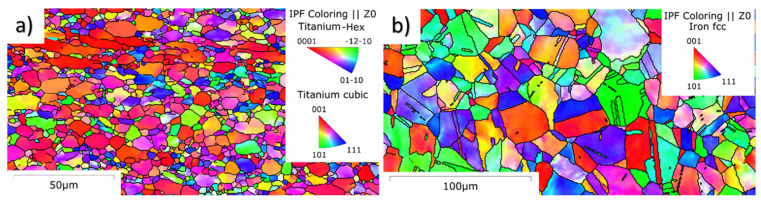
EBSD analysis of transversal cuts of samples of (**a**) Ti6Al4V and (**b**) 316L.

Two experimental materials were used in the experiment. Austenitic stainless steel 316L was chosen due to its erosion resistance. The material has a face-centered cubic lattice. The second material was titanium alloy Ti6Al4V, which is widely used material in number of bioapplications. This material consist of alpha HCP phase and beta BCC phase. Therefore, pure water jet peening and cutting are attractive for various applications. The average grain orientation of 316L determined by EBSD is shown in Figure 1. The random grain orientation of both materials is confirmed in Figure 1, and grain size and phase composition determined by EBSD are listed in Table 1. Chemical composition is listed in the Table 2 and Table 3.

Samples for the PWJ experiment and the following microscopical observation were cut using a precision abrasive cutter (Brilliant 220, ATM, Mammelzen, Germany) with a constant flow of cooling liquid. Cooling liquid was based on water with the addition of antibacterial alkaline additive Blasorun 5 and ATM-CoolCut (ATM GmbH, Mammelzen, Germany). All samples were then sequentially ground using abrasive papers with average grain sizes of 46, 22, 18, and 15 µm. After mechanical grinding, fine polishing was applied. For the austenitic steel samples, electrolytic polishing was used with the following parameters: 0 °C, 15 s, and 20 V in a solution consisting of ethanol, perchloric acid, and nitric acid. The titanium samples were polished using diamond paste with grain sizes of 3 and 1 µm followed by a vibratory polisher (Vibromet 2, Buehler, USA). The polished samples were subjected to water jet treatment for increasing durations.

**Figure 2:**
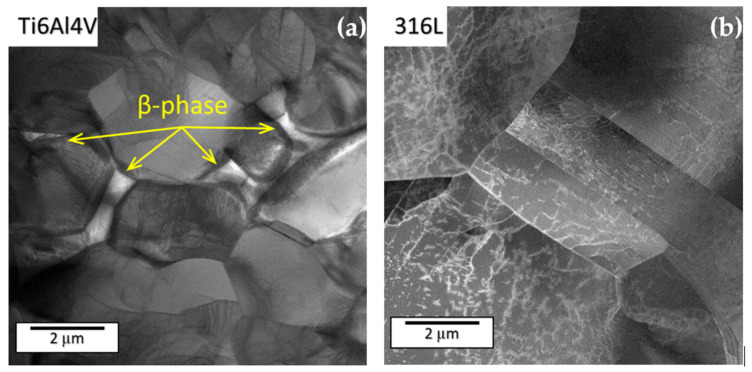
TEM observation of as-received (**a**) Ti6Al4V and (**b**) 316L.

Figure 2 depicts TEM (JEOL, Tokyo, Japan) images of both materials in the as-received state. Dislocation density in Ti6Al4V is quite low, the magnitude is 10^7^ m^−2^. There was a number of pile-ups on the α/β interface. The β phase was visible on the grain boundaries of α. In the as-received state of the 316L steel, dislocations from multiple slip systems form nets in the interior of the grains and frequent pile ups close to the boundaries. The dislocation density is variable with the magnitude of order 10^11^ m^−2^. The material was composed of an FCC matrix with small number of bands of residual delta ferrite. No mechanical twins or epsilon martensite were observed.

## 3. Experiments

**Figure 3:**
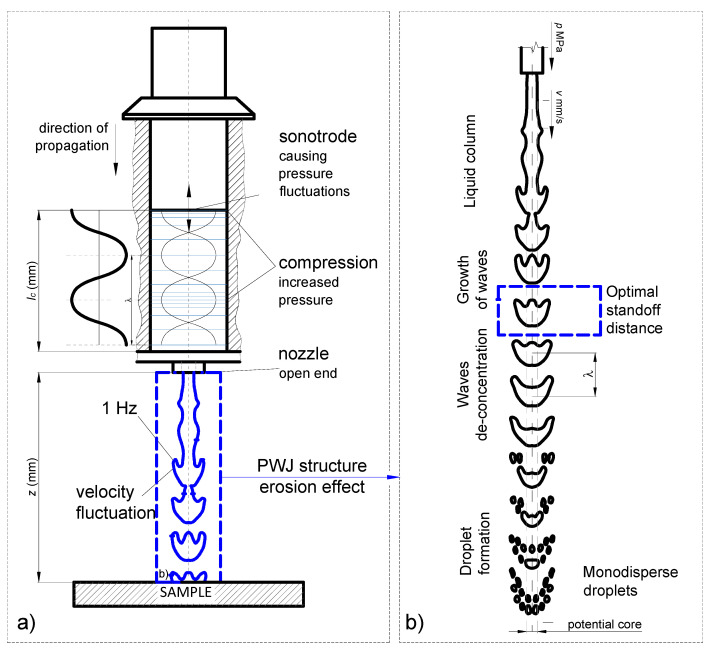
Experimental setup: (**a**) schematic figure of the device; (**b**) standoff distance estimation for water droplet use.

The samples were treated using PWJ technology. The equipment was located in the Institute of Geonics of CAS in Ostrava, Czech Republic. The surface of the sample was treated by an increasing number of impingements. The number of impingements was defined by the set of several levels of running times of the sonotrode (Ecoson, Nove mesto and Vahom, Slovakia). The time periods ranged from 0.05 to 3 s, which correlate with 2100 to 63,000 impacts, respectively. As the source of pressurized water, a Hammelmann HDP 253 pump (Hammelmann, Oelde, Germany) was used. It could reach an operating pressure of up to 160 MPa and flow up to 67 L/min. The source of pressure pulses was an ultrasonic generator (Ecoson WJ-UG-630-40) (Ecoson, Nove mesto and Vahom, Slovakia) connected to a piezoelectric generator. The power output of the ultrasonic system could reach up to 800 W. The length of the acoustic chamber was set to 12 mm; the frequency of the sonotrode was set to 21 kHz; the standoff distance was set to 5.5 mm. The process parameters are listed in Table 4. Based on the comparison of pressures used in this experiment with pressures used in the literature, the erosion process was expected to be of low intensity, i.e., only the initial stages of the erosion process should be reached. During experiments, the PWJ head was positioned stationary over the treated area. The positioning of the PWJ head was controlled by a robotic arm (ABB IRB 6640-180/2.55) (ABB, Zürich, Switzerland). The schematic of the experiment is depicted in Figure 3.

The number of impingements for each run of nozzle (StoneAge, Durango, CO, USA) velocity *v* = 0 mm/s was calculated according to:

(1) $$n_{i}=f.t$$

### 3.1. Measurements

The first step of material observation was to detect the surface irregularities caused by the increasing number of impingements created by the PWJ using a confocal microscope (LEXT OLS 3100 microscope, Olympus, Tokyo, Japan). For height, the profile height map of the completely impacted surface (magnification ×240, view field 1280 × 960 µm) was used. A magnification ×1200 (view field 256 × 192 µm) was applied to measure the height of the primary profile (Pz) at selected regions of the impinged area. The raw 3D image was subjected to spike removal in order to remove isolated pixels (Pixel count 3) in Lext OLS software (Olympus, Tokyo, Japan). The data were also leveled by mean plane substraction. A Tescan Lyra3 XMU FEG/SEMxFIB (Tescan, Brno, Czech Republic) scanning electron microscope was employed to observe the impacted surface and surface irregularities, as well as for the EBSD analysis (Oxford Instruments, Abingdon-on-Thames, Great Britain). The focused ion beam (FIB) technique available for the Tescan Lyra3 SEM was used for the preparation of lamellas for transmission electron microscopy (TEM); the conditions for lamellas preparations using the FIB were 30 kV voltage and 10^−4^ nA current. The TEM samples from areas with surface irregularities were observed to assess the changes in dislocation structure caused by the periodically acting pulses with increasing numbers of impingements. The TEM foils of the as-received materials were prepared by electrolytical polishing. TEM observation was conducted using a JEOL JEM-2100F microscope (Jeol, Tokyo, Japan).

### 3.2. Results

#### 3.2.1. Confocal Microscopy of Ti6Al4V

**Figure 4:**
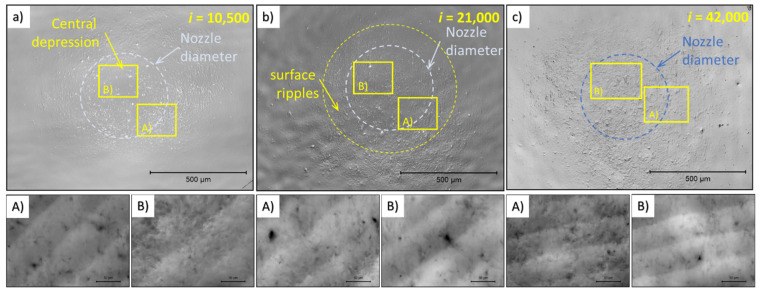
Results of confocal microscopy showing the evolution of erosion damage to the polished surface of titanium with increasing the cumulative impingements (**a**) is 10,500 impingements, (**b**) is 21,000 impingements and (**c**) is 42,impingements. The impingements are from a stationary source using an ultrasonic pulsating water jet with an impact velocity of 150 m/s and water cluster diameter d of 0.4 mm, indicated by the dashed line.

**Figure 5:**
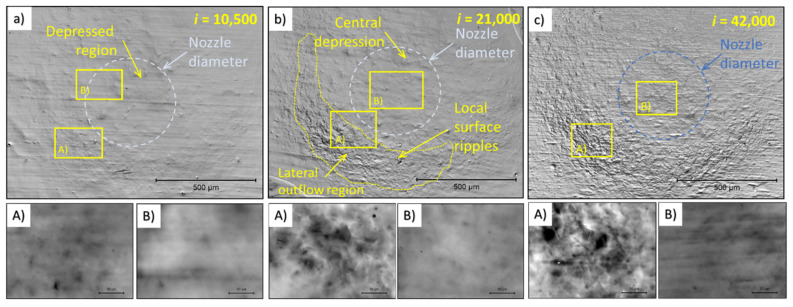
Results of confocal microscopy showing the evolution of erosion damage to the polished surface of AISI 316L stainless steel with increasing cumulative impingement numbers (**a**) is 10,500 impingements, (**b**) is 21,000 impingements and (**c**) is 42,impingements. The impingements are from a stationary source using an ultrasonic pulsating water jet with an impact velocity of 150 m/s and water cluster diameter *d* of 0.4 mm, indicated by the dashed line.

Confocal microscopy (Olympus, Tokyo, Japan) was used to detect surface irregularities caused by the PWJ. The results obtained on the Ti6Al4V samples are presented in Figure 4 for different durations of PWJ application, i.e., for increasing numbers of impingements. In the Figure 4 and Figure 5, an overview picture is provided of the dot created by the PWJ, together with the rectangles in locations A and B of further analyses in the form of micrographs, height profiles of evaluated parameters of the height of the primary profile. To determine height, the profile height maps of the completely impacted surface at magnification ×240 (view field 1280 × 960 µm) were used. For measurement of the height of the primary profile at selected areas of the impinged area, a magnification ×1200 (view field 256 × 192 µm) was used. Area B (shown in the overview picture) is inside the nozzle diameter; area A is located in the areas with the highest showing most significant deformation. The area impacted by 10,500 impacts is shown in Figure 4. The high magnification of area B shows a wavy surface created by the preparation of the sample. At a high magnification of area A, after 10,500 impacts, the disintegration of the original surface was observed due to the angling of the grains, making the shape of the grains visible even on the height profile. After doubling the number of impacts, further grain tilting was observed along with a number of spots with a considerably lower profile (beginning of micro-pits). Figure 4 shows a Ti6Al4V sample treated with 42,000 impacts. In the area, strong grain tilting was observed. Moreover, a number of spots and lines with a lower profile was observed; these locations, with further impingements, may develop into stress concentrators, then depressions, and then micro-pits, finally being the source of localized erosion.

#### 3.2.2. Confocal Microscopy of AISI 316L

**Figure 6:**
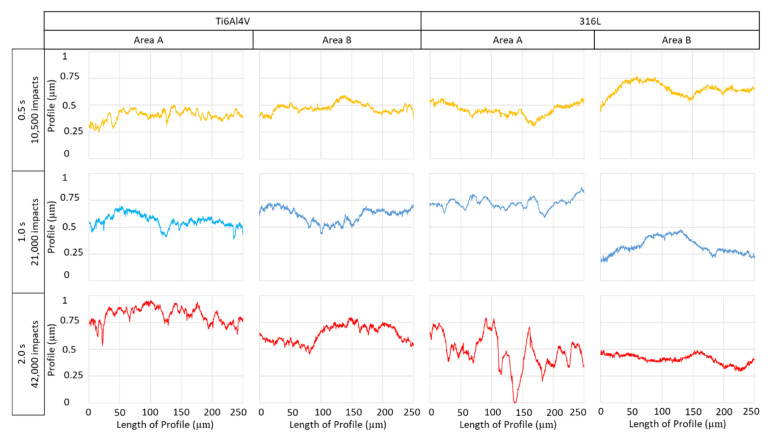
Results of confocal microscopy of Ti6Al4V and 316L samples treated by 42,000 impacts from a pulsing water jet (PWJ), supported by a graph comparing the profiles of the different number of impacts for areas A and B.

The 316L samples showed much faster development of the height profile with increasing numbers of impacts. After 10,500 impingements (Figure 5), the low magnification picture showed an area with occasional waviness, and in area A, there were some signs of twins, grain tilting, and local depressions. These effects are hard to observe from the low magnification image after 10,500 impacts. The difference between irregularities caused by sample preparation (shallow perpendicular lines) and irregularities caused by the PWJ was clear even after 10,500 impacts. Increasing the number of impacts to 21,000, the surface, at low magnification, showed a significant increase in irregularity between areas A and B. The detail of area B shows a relatively flat surface, whereas area A shows clearly visible grain boundaries and a number of sharp steps inside the grains. Figure 5 shows the area impacted by 42,000 water droplets, more clearly depicting the shape of grains (grain tilting) and a higher number of sharp steps. The differences between the details of areas A and B are even more significant. The area with a taller height in the primary profile has a crescent moon shape, which might be explained from the process point of view, as well as by the material response. The crescent shape could indicate nonuniform velocity distribution across the cross-section of the water droplet/cluster. From the material perspective, the material directly under the jet can accommodate some of the impact pressure by elastic deformation, which then travels through the material in a limited range, causing plastic deformation in the adjacent area. The differences with increasing number of impacts between area A and area B are shown in Figure 6. Comparing the surface after 42,000 impacts of 316L (Figure 5) and Ti6Al4V (Figure 4), 316L showed a higher amount of roughening, nonuniformly spread across the impacted area (crescent shape). The 316L samples also showed areas with a lower profile, known as depressions, whereas the depressions on Ti6Al4V focused more in points or lines.

Figure 6 compares the different profiles taken from areas A and B depicted in the details in Figure 4 and Figure 5 with increasing numbers of impingements. The graph shows similar development of the surface profile in the micrographs. The sharp depressions were the most apparent for area A on 316L stainless steel treated with 42,000 impacts. Table 5 shows measured properties of primary profiles from selected areas.

**Figure 7:**
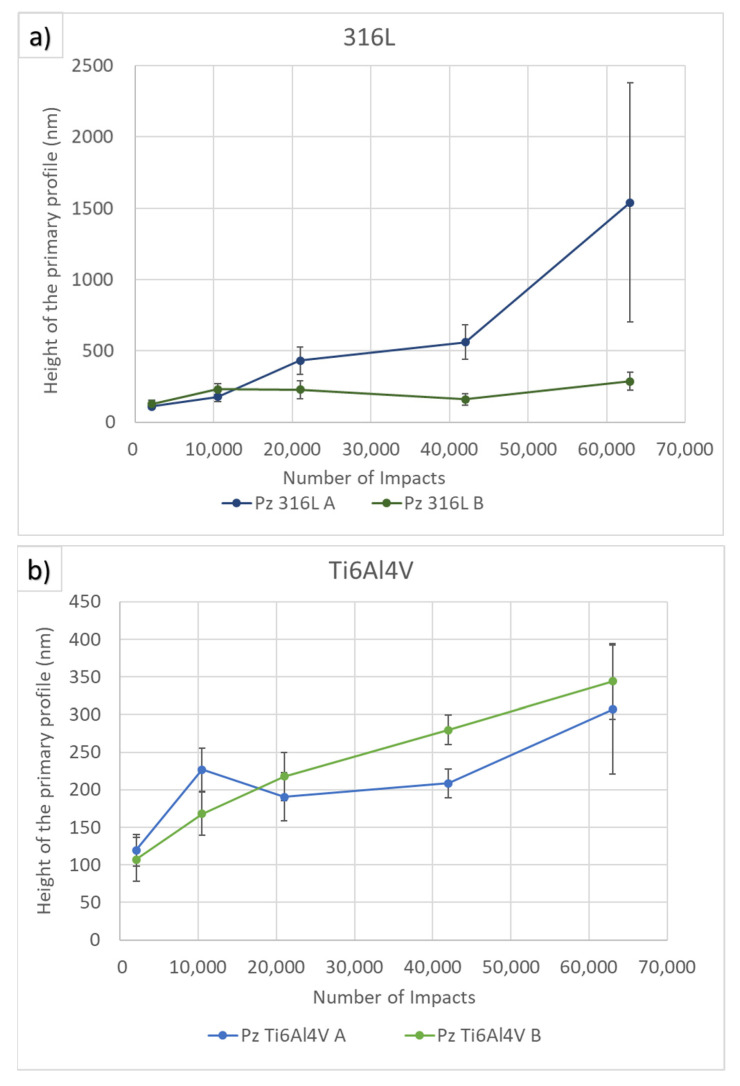
The relationship of primary profile parameter Pz with number of impacts. (**a**) shows evolution in austenitic stainless steel 316L and (**b**) shows evolution in two phase titanium alloy Ti6Al4V.

The evolution of the height of the primary profile (Pz) with duration of PWJ treatment was evaluated from detailed confocal images (view field 256 × 192 µm). The results were averaged from five measurements. The average values of the results together with scatter bars are shown in Figure 7**.** The plots show the increase in Pz with increasing the number of impacts from 1050 to 63,000 for area A (area with the tallest height in the primary profile) and area B (center of the impacted area). The trends for both materials, 316L and Ti6Al4V, were similar. In the case of Ti alloy, the increases in primary profile maximum height for both areas A and B were similar, i.e., approximately 200 nm. Contrary to Ti6Al4V, 316L showed a fast increase in area A (approximately 1400 nm) and a steady increase in area B (approximately 200 nm). With increasing the number of impingements, the difference in Pz became more prominent in 316L. We observed an insignificant difference between areas A and B after a small number of impingements (up to 21,000). With 42,000 impingements, area B had a roughly 2.5 times higher Pz than area A. For 63,000 impingements, the height of the primary profile difference was five times greater.

#### 3.2.3. SEM Results for Ti6Al4V

**Figure 8:**
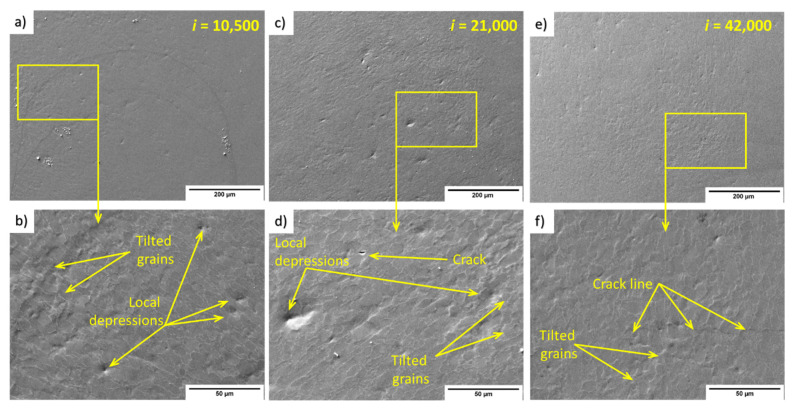
SEM micrographs of the surface of a Ti6Al4V sample treated by PWJ for different durations, all in SE mode: (**a**) Overview and (**b**) detail of the surface after 10,500 impacts, (**c**) Overview and (**d**) detail of the surface after 21,000 impacts, (**e**) Overview and (**f**) detail of the surface after 42,000 impacts.

Figure 8 shows an overview and the details of the impacted areas after 10,500, 21,000, and 42,000 impacts. The 10,500 impacts produced a wavy area on the left side, where the edges of the waves copy the interfaces of the α/β phases. The detail also shows four visible point−focused depressions. After 21,000 impacts, a wavy area with a number of depressions was observed, as shown in the magnified area. In the top left quartile of the high-magnification picture (Figure 8b), a crack can be observed at the edge of the α/β phases.

#### 3.2.4. SEM Results for 316L

**Figure 9:**
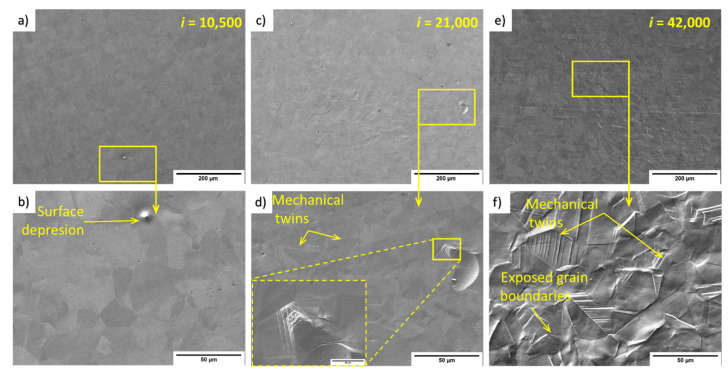
SEM micrographs of the surface of a 316L sample treated by PWJ for different durations, all in SE mode. (**a**) Overview and (**b**) detail of the surface after 10,500 impacts. (**c**) Overview and (**d**) detail of the surface after 21,000 impacts. (**e**) Overview and (**f**) detail of the surface after 42,000 impacts.

Figure 9 shows SEM images of 316L impacted by 10,500, 21,000, and 42,000 water volume droplets. The detail for 10,500 droplets in Figure 9a shows a very low number of visible erosion and deformation signs. Sporadic depressions are depicted at the top edge of the detailed image. After 21,000 impacts (Figure 9b), the number of grains with convenient orientation shows sharp steps, visible from the detailed SEM image. On the right side of the image is a strange formation over several grains. Closer observation shows that the formation is not a particle, but rather a pit or crater of approximately 20 μm. It was most likely created (1) as a kind of impact to the surface during surface preparation prior to the PWJ treatment, and then PWJ formed twins from the pit to the grain boundary in the vicinity or at the front of the dimple, or (2) by some dust or sand particle as an accidental admixture, and, again, the formation of twins occurred from the pit and ended at the grain boundary. The area after 42,000 impacts showed a high number of grains with sharp steps visible even from the overview (top) SEM figure. As shown in Figure 9, the tilting of grain boundaries led to depressions and upheaval. The mechanical twins’ evolution from 21,000 (Figure 9e) to 42,000 impacts (Figure 9f) is also noteworthy. By doubling the number of impingements (active time), the mechanical twins became noticeably more exposed.

#### 3.2.5. TEM Results for Ti6Al4V

**Figure 10:**
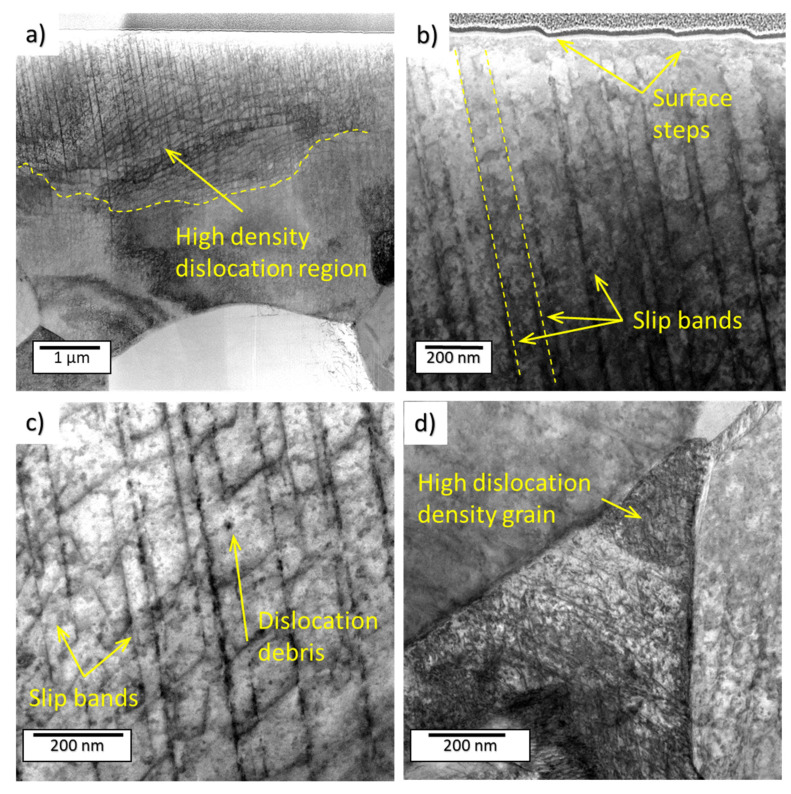
Micrographs of Ti6Al4V treated with 52,500 impacts. Scanning transmission electron imaging (STEM) in bright field (BF) mode. (**a**) Pt layer is seen at the upper part of the micrograph. High dislocation density just below the surface is clearly seen. (**b**) Surface steps correlates with slip bands in the interior of the grain.(**c**) Slip bands and dislocation debris in the interior of the grain.(**d**) Dense dislocations net in a suitably oriented grain.

TEM micrographs of the Ti6AL4V alloy from the sample treated with the PWJ for 2.5 s are shown in Figure 10. The figure depicts the microstructure under the center of the impingement area close to the surface. The TEM lamella was extracted using the FIB milling perpendicular to the surface. The original surface of the material was covered by a layer of amorphous Pt to protect the surface relief, produced by the water impingements, from damage by the Ga ions. Figure 10a reveals a grain with high dislocation density close to the surface. Dislocation density was measured on TEM micrographs using the line intercept method according to the formula ρ = 2N/Lt according to [29], where N is number of intersections between randomly drawn grid lines on the micrograph with dislocations, L is total length of lines, and t is the foil thickness. The densities were measured on the FIB lamellas, so the inspected area was rather small, typically 5×10 µm. The foil thickness was measured during fabrication of FIB lamellas with precision better than 10%.

The dislocations were regularly distributed in the grain; moreover, parallel slip bands with higher dislocation density were observed. It seems that the dislocation density ρ was the highest just under the surface (ρ = 5 × 10^14^ m^−2^) and decreased slightly with distance from the surface. Figure 10b,c shows an array of slip bands along the crystallographic slip plane. When the slip bands reached the surface, inclined surface steps were formed. The bands were more or less regularly spaced (distance between bands was about 150 nm in the observed grain). They were formed by dislocations with high density (Figure 10c). The surface steps showed intense slip activity along the bands; intense dislocation multiplication quite probably occurred along the bands. The dislocation debris (small dots and probably prismatic dislocation loops) can be observed. We were unable to determine if the debris was the product of plastic deformation or an artefact of ion milling. A grain with very high dislocation density is shown in Figure 10d. The dislocation density in this grain was estimated as ρ = 3 × 10^15^ m^−2^.

#### 3.2.6. TEM Results for 316L

**Figure 11:**
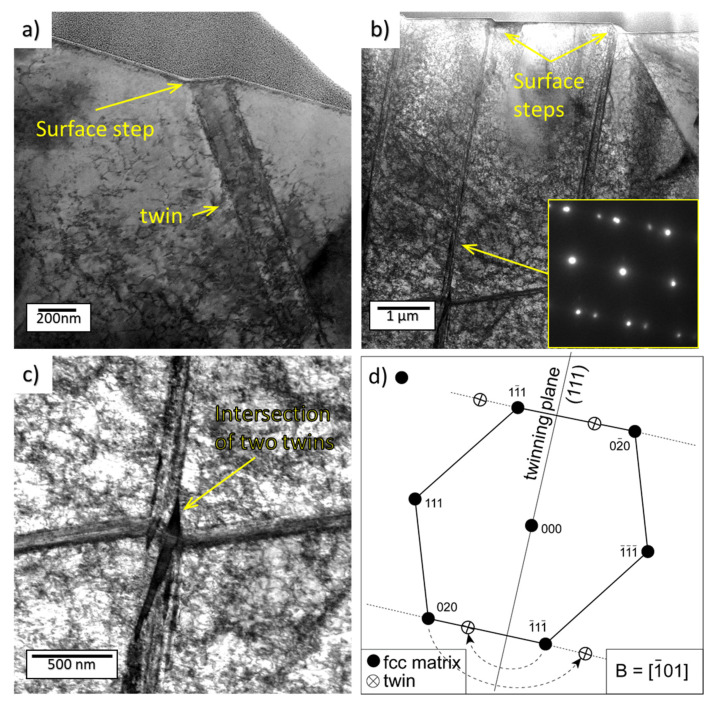
TEM micrographs after (**a**) 52,500 and (**b**,**c**) 57,750 impacts, all in BF mode, and (**d**) schema of twinning.

The microstructure formed by the PWJ under the impinged surface was studied in austenitic steel (Figure 11). The dislocation density in the observed area was again very high, about 2 × 10^14^ m^−2^, which is much higher than common values after tensile or fatigue testing. Dislocations did not rearrange into some 3D regular structures as was observed in fatigued samples [30] ; repeated water impingement thus has a different effect on the microstructural evolution compared with mechanical fatigue tests. The volume of the specimen was regularly filled with dislocations with a weak tendency to form dislocation walls. A band about 200 nm thick reaching the surface is visible in Figure 11a. An inclined step formed at the surface, which corresponded to the surface bands observed, e.g., in Figure 9f. Several similar thinner bands are shown in Figure 11b. One system of such bands intersects the surface with a sharp angle, while there is a band almost parallel to the surface. The analysis of the diffraction diagram from the matrix and a selected band (in Figure 11b) proved that the bands were thin mechanical twins with the {111} twinning plane. Detail of the cross−section of the two twins is shown in Figure 11c. The schematics of the indexed diffraction pattern in Figure 11d show the orientation of the twinning plane and the origin of the extra spots due to twinning. The mechanical twins with the {111} twinning plane and Σ3 boundary are the most usual twins appearing in FCC materials.

## 4. Discussion

In this study, the surfaces of two different materials with different crystallographies (316L and Ti6Al4V) treated with increasing numbers of impingements were examined using SEM. The height values of surface irregularities were measured using confocal microscopy. Beneficially, we observed the changes in dislocation arrangement under the treated surface using TEM.

From the TEM micrographs, we performed a detailed evaluation of the processes occurring inside the materials in the first erosion stage. In contrast to the 1970 work of Thomas and Brunton [14], who divided the erosion process into three indicative stages, Foldyna et al. [12] described these stages as: (1) plastic deformation, (2) creation of erosion pits, and (3) merging of erosion pits into a single crater. This distribution is based on surface observation and measurements of primary profile and depth. These stages provide a good basis for understanding the erosion processes. Figure 8 and Figure 9 show the transition mechanism between the first and second erosion stages that occurred after doubling the number of impingements. The main mechanism is the creation of depressions by grain tilting and intense growth of twins or slip bands.

However, to predict the erosion process in advance and to use water impact for surface strengthening, it is necessary to describe and divide the first stage of plastic deformation into separate stages. Several methods have been used for this purpose, mainly in conjunction with PWJs, mostly using surface hardening as the measured property. These methods mainly include residual stresses [15] as well as microhardness [18]. In this article, the focus was the observation of changes in the dislocation structure correlated with surface irregularities. It is well−known that mechanical hardening causes increases in dislocation density [11,15]. The increase in dislocation density, according to Taylor’s equation [31], causes increase in the internal stresses and, in turn, hardening of the subsurface layer. The values of dislocation densities under the surface, given in the Results section, are locally precise, as the thickness of the FIB foil is known [29]. However, it is obvious that the dislocation density fluctuates in the subsurface layer and due to the limited area of FIB foils, no statistics of dislocation density fluctuation could be calculated. Based on the measurements in [32], the relative error of given values could be as high as 50%.

Our confocal study showed an increasing height of the primary profile (Pz) with increasing numbers of impacts. The results of the confocal study showed that tilting of the grains is the primary mode of plastic deformation for both materials; however, it is more visible in 316L. This tilting leads to the creation of depressions, which are thought to be sources of micro-pits. The characteristics of these depressions differ between Ti6Al4V and 316L, as the depressions of 316L span across a larger area. The tilting of the grains is also accompanied by accommodating mechanisms inside the grains, as described in the TEM sections. Importantly, the height profiles study and the Pz evolution graphs showed that, with increasing numbers of impingements, a sharp occurs on the edges of the impacted area compared with the middle of the area. This was especially apparent on 316L, where the highly roughened area was apparently a crescent moon shape. This could have been caused by several factors, the first of which is the process which indicated nonuniform velocity distribution across the water droplet/cluster. The second possibility is the elastic/plastic response of the material.

SEM images showed the creation of point depressions on Ti6Al4V, which evolved into area depressions with increasing impingements. Notably, these depressions are often created on the edge of α grains. The 316L samples showed many sharper steps; however, grain tilting was not as apparent compared with Ti6Al4V. In this case, sharp steps can also provide depression locations for micro-pits initiation.

Most of the researchers expect the dislocation structure created by PWJs to be similar to that created mechanically by cyclic deformation due to the similarity of surface irregularities between the fatigued sample and the early−erosion−stage sample. However, this TEM study showed that the subsurface layers of the materials underwent intense plastic deformation, which resulted in surface roughening. The dislocation density close to the surface was very high, but the dislocations did not rearrange in low−energy dislocation structures/patterns as usual in fatigued samples, where, e.g., ladder, labyrinth, or cell structures were reported [33,34].

The dislocation structure evolution in material treated by PWJs still needs investigation. Most of the researchers expect the dislocation structure created by PWJs to be similar to that created by fatigue, due to the similarity of surface irregularities between the fatigued sample and the early−erosion−stage sample. The TEM study showed that the subsurface layers of the materials underwent intense plastic deformation, which resulted in surface roughening. The dislocation density close to the surface was very high, but the dislocations did not rearrange in low-energy dislocation structures/patterns as usual in fatigued samples. Whereas surface roughening of Ti6Al4V was mainly caused by grain tilting, the surface steps observed in α grains were proved by TEM to be slip bands. Surface roughening of 316L, at least during the early stages in this study, was caused primarily by intense mechanical twinning and not by dislocation slip. The same type of 316L steel was used for fatigue testing and a microstructural study. The mechanical twins were observed in the specimens fatigued to fracture; however, they were rare. It seems that after PWJ, the density of twins and dislocation density were high (2 × 10^14^). This was a consequence of the very high peak stresses induced in the material by the water hammer effect at the moment of contact of a water drop with the surface. This stress pulse was very short, but was enough for nucleation, propagation, and growth of twins. It is generally accepted that twinning is a stress-induced effect. Therefore, the high stress peak can induce twins even in materials that are not prone to twinning in mechanical testing, as the stress reached in tensile or fatigue tests is not high enough for twin nucleation. Therefore, the high stress peak can induce twins even in materials that are not prone to twinning in mechanical testing, as the stress reached in tensile or fatigue tests is not high enough for twin nucleation. The appearance of twins demonstrates again that the acting of repeated forces due to the (1) mechanical testing and (2) impacts of water droplets have different consequences in the microstructural evolution.

## 5. Conclusions

Liquid droplet impingement on polished surface of stainless steel and titanium alloy was experimentally studied using ultrasonic water jet as a droplet generator. By changing the time exposure, the number of impacts of water droplets were controlled in order to observe the erosion damage evolution focusing on early erosion stages with following results:

- The surface profile, quantitatively measured by confocal microscopy and characterized by the height of the primary profile of surface, is more intense in austenitic stainless steel than in Ti alloy; the difference between the two materials increases with the number of impacts and is five times higher with 60,000 impingements.
- The surface of the Ti alloy in the incubation stage can be described as wavy, with local depressions and short cracks formed preferably at the interface between the α and β phases.
- The surface of the 316L austenitic stainless steel shows sharp parallel slip lines along crystallographic planes within individual grains. With the increasing water droplet impingement, a significant increase in the height of the primary profile was observed. The twinning and intense grain tilting were observed as the main mechanisms.
- The microstructural changes due to the repeated short, localized impacts of water droplets is different from those induced by mechanical fatigue. The dislocation density in the subsurface layer with typical thickness about 5 µm of both studied materials increased by several magnitudes up to 1014–1015 m^−2^. These values are so high that the capacity of material to accommodate plastic deformation seems to be exhausted and the formation of cracks follow. Dense slip bands of dislocation are observed in the Ti alloy, probably along the planes where dislocation sources were activated. In 316L steel, the dislocations are homogeneously distributed without signs of rearrangement in, e.g., cell structures, frequently observed in fatigued specimen. The reason lies in short, localized pulses of stresses due to the impingement, when dislocations are mobile only in a limited volume for a short time. Moreover, intense mechanical twinning observed in 316L, which is also unusual in specimens subjected to mechanical tests, is the reason for the pronounced surface steps and lines.
- Future direction research will be oriented in expanding the knowledge dealing with erosion resistance of materials focusing on subsurface damage in early erosion stages in an engineering material.

## Figures and Tables

**Table 1:** Mechanical Properties of Experimental Materials.

Material	Hardness HV0.2	Elasticity Modulus (GPa)	Tensile Proof Stress R_p_0.2 (MPa)	Tensile Ultimate Strength R_m_ (MPa)	Average Grain Size (µm)	Phase Composition
AISI 316L	184 ± 10	198	322	625	10.2 ± 6.7	100% Austenite FCC
Ti6Al4V	372 ± 6	123	936	1036	3.4 ± 2.4	98% Ti HCP (α) 2% Ti BCC (β)

**Table 2:** Chemical Composition of AISI 316L %wt.

**AISI 316L**	**C**	**Cr**	**Mn**	**Mo**	**N**	**Ni**	**Si**	**Fe**
	0.02	16.63	1.26	2.04	0.04	10.00	0.38	bal.

**Table 3:** Chemical Composition of TI6Al4V %wt.

**Ti6Al4V**	**C_max_**	**Fe_max_**	**N_max_**	**Al**	**O_max_**	**V**	**H_max_**	**Y_max_**	**Other**
	0.08	0.03	0.05	5.50–6.75	0.20	3.5–4.5	0.015	0.005	0.40

**Table 4:** Technological conditions of the experiment.

Run	Pressure *p* (MPa)	Frequency *f* (kHz)	Chamber Length *lc* (mm)	Nozzle Diameter *d* (mm)	Standoff Distance *z* (mm)	Flow Speed *v_w_* (m/s)	Flow Rate *m* (m/s)	Time Exposure *t* (s)	Number of Impacts (-)
1	15	21	12	0.4	5.5	156.04	1.18	0.05	1050
2								0.1	2100
3								0.25	5250
4								0.5	10,500
5								0.75	15,750
6								1	21,000
7								1.25	26,250
8								1.5	31,500
9								1.75	36,750
10								2	42,000
11								2.25	47,250
12								2.5	52,500
13								2.75	57,750
14								3	63,000

**Table 5:** Surface profile parameters measured in areas A and B.

Area	Roughened Area A					Middle Area of the Impacted Region B				
Number of impacts	2100	10,500	21,000	42,000	63,000	2100	10,500	21,000	42,000	63,000
Material	316L									
Primary profile value (Pa)	31.14 ± 4.33	47.04 ± 8.09	90.28 ± 17.83	122.48 ± 14.42	313.86 ± 142.97	33.42 ± 7.35	46.07 ± 8.67	44.25 ± 15.41	40.18 ± 13.94	57.52 ± 11.93
Primary profile skewness (Psk)	−0.25 ± 0.49	0.10 ± 0.35	−0.29 ± 0.50	−0.12 ± 0.41	0.29 ± 0.70	−0.01 ± 0.48	−0.08 ± 0.44	−0.04 ± 0.41	−0.29 ± 0.45	−0.04 ± 0.27
Primary profile kurtosis (Pku)	2.59 ± 0.71	2.89 ± 0.84	2.87 ± 1.25	3.05 ± 1.11	3.32 ± 1.21	2.43 ± 0.36	2.68 ± 0.50	2.64 ± 0.57	2.83 ± 0.89	2.38 ± 0.37
Material	Ti6Al4V									
Primary profile value (Pa)	32.96 ± 8.53	46.13 ± 7.51	55.70 ± 14.27	45.41 ± 5.66	59.82 ± 26.49	29.96 ± 6.23	38.11 ± 4.23	65.22 ± 10.36	84.32 ± 20.48	60.35 ± 13.56
Primary profile skewness (Psk)	0.31 ± 0.51	0.10 ± 0.63	−0.03 ± 0.53	0.01 ± 0.57	−0.03 ± 0.40	0.29 ± 0.35	−0.45 ± 0.38	−0.19 ± 0.70	0.16 ± 0.29	−0.07 ± 0.27
Primary profile kurtosis (Pku)	2.55 ± 0.48	3.21 ± 0.66	2.63 ± 0.28	2.92 ± 0.69	3.18 ± 0.92	2.89 ± 0.78	3.34 ± 0.90	2.66 ± 0.59	1.85 ± 0.30	2.70 ± 0.45
